# Experimental Analysis of the Force Stresses on the Protrusions of Profile Conveyor Belts Using a Sensor

**DOI:** 10.3390/s26041353

**Published:** 2026-02-20

**Authors:** Leopold Hrabovský, Lucie Vlčková, Jan Blata, Ladislav Kovář

**Affiliations:** Department of Machine and Industrial Design, Faculty of Mechanical Engineering, VSB Technical University of Ostrava, 17. listopadu 2172/15, Poruba, 708 00 Ostrava, Czech Republic

**Keywords:** steep conveyor belt transport, profiled conveyor belt, rib, shear friction coefficient, angle of inclination of the protrusion

## Abstract

Profile conveyor belts are used in operational applications where the transport of bulk materials is required at high inclinations on conveyor belts, typically in the range of 30–40°. This paper deals with the analytical determination of the critical angle of inclination of a homogeneous transverse profile (protrusion), beyond which relative movement of bulk material occurs on the surface of the conveyor belt. The compressive forces induced by the known gravity component of the bulk material acting on a 20 mm high transverse protrusion were experimentally measured on a specially designed laboratory apparatus. The measurements were performed at different inclination angles of the folding plate, which simulated the working surface of the conveyor belt. During the experiments, the investigated bulk material—river gravel with a grain size of 4 ÷ 8 mm—was placed in a plastic frame with a width corresponding to the defined loading width of the conveyor belt. On the basis of the measured values of compressive forces, the static coefficient of shear friction in contact with grains of bulk material with two types of surfaces, namely plastic and rubber, was analytically determined. From the experimental data, the mean values of the static shear friction coefficient were determined, which were 0.33 for the plastic surface and 0.48 for the rubber surface, with the orientation of the protrusion perpendicular (90 deg) to the longitudinal axis of the conveyor belt. The experimental investigation also included the determination of the internal friction angle of the river gravel. The results show that when bulk material is conveyed by a profile conveyor belt, it is possible to safely convey material with a cross-sectional height greater than the height of the transverse protrusion, provided that the conveyor inclination angle does not exceed the internal friction angle of the bulk material.

## 1. Introduction

Belt conveyors [1] are continuously operating conveying devices [2] designed for the continuous transport of bulk and piece materials by means of an endless loop of a pulling element, which is a conveyor belt. The advantages of belt conveyors are smooth transport with a high conveying capacity [3,4], suitability for the transport of virtually all bulk materials, low movement resistance [5,6,7,8,9], noiseless operation, safe and reliable operation [10,11], and simple construction with easy assembly and disassembly. The disadvantages are the large number of rotating parts, the difficulty of transporting abrasive and adhesive materials, and the limited angle of inclination of the transport [12,13,14].

Authors Zrnić et al. discuss the history of belt conveyors over the years, up to the development of the modern belt conveyor at the end of the 19th century in papers [15,16].

Thomas Robns Jr and the Robins Conveying Belt Company published the first known belt engineering handbook in 1917, “Handbook of Conveyor Practice” [17].

The conveyor belt is one of the basic parts of the belt conveyor; it forms an endless element orbiting around the end drums, performs the function of carrying the conveyed material, and at the same time performs the function of the tensile element, which transfers all the movement resistance [18,19] arising during its circulation [20].

A relatively large number of papers are devoted to the area of damage causes, e.g., [21,22], and joint diagnosis, e.g., [23,24], of conveyor belts. From the research in different articles, it can be concluded that the amount of material profile conveyor belts are able to convey at transport inclination angles up to 40 deg has not been investigated in detail. In this paper, the authors are concerned with the detection of the applied compressive force on homogeneous profiles, which are named “protrusions”, of profile conveyor belts at different transport inclination angles. This paper also defines the cross-section of the belt filling $A_{1}$ [m^2^] (whose height exceeds the height of the protrusion) and the volume $V_{1}$ [m^3^] that can be safely transported at a given transport inclination angle $\delta_{1}$ [deg] and known mechanical–physical parameters of the transported material (grain size, dynamic angle of repose [25], coefficient of internal friction [26], etc.).

In paper [27], the authors P. Banerjee et al. investigate a machine vision-based technique that is specifically designed for assessing surface wear on cleated conveyor belts across the width and belt thickness.

For belt conveyors of classic construction, conveyor belts [28] with a supporting frame made of textile inserts (P—polyamide, E—polyester, V—viscose, Pvs—polyamide + viscose shear, etc.) or steel ropes [29] (for transferring larger tensions) are used. Conveyor belts with a textile frame [30] consist of a frame consisting of inserts, which is protected on both sides by cover layers and protective edges. In very difficult working conditions, conveyor belts are used whose frame is protected from damage by a bumper [31].

For belt conveyors of special designs [32,33], including steep [34,35] and vertical [36] belt conveyors, structurally modified conveyor belts are used. The design modifications of conveyor belts enable the conveying of bulk materials at inclination angles that exceed the limiting inclination angle of conveying by belt conveyors of conventional design [18]. In practice, profiled conveyor belts and cleated conveyor belts are widespread [32,37,38,39].

Profile conveyor belts [40,41,42] are used for steep conveying [32,34] of bulk materials with an inclination angle of the conveying route of max. 40 deg. The protrusions on the working surface of a WALTER [43] or CHEVRON [44] profile belt are vulcanized at the same time as the belt, while the protrusions (homogeneous profiles) are part of the cover layer.

The lack of a uniform methodology or practical guidance for determining the belt filling cross-section of profile conveyor belts represents a significant complication in practice when theoretically designing the conveying capacity of these steep belt conveyors. This article defines the basic parameters that must be considered when assessing the failure-free transport of bulk materials by profile conveyor belts.

A completely new and so-far-unpublished solution is the analytical determination of the size of the angle of inclination of the protrusions $\alpha_{1}\;[\deg]$ of profile conveyor belts, at which the relative movement of the conveyed grains of bulk material occurs in the direction of the width of the conveyor belt.

In the case of profile conveyor belts [43,44], the optimal design of the shape and the angle of inclination of the protrusions $\alpha_{1}$ [deg] can prevent the grains of the bulk material from being conveyed and spilling over the edges of the conveyor belt. If the shape of the protrusions is chosen so that its end parts are parallel to the longitudinal axis of the conveyor belt, then it is ensured that the conveyed material grains will be spread on the conveyor belt over the used loading width b [m]. If the protrusions are inclined to the longitudinal conveyor belt at an angle greater than the $\alpha_{1}$ [deg] angle, then the material grains will tend to move toward the center of the conveyor belt width.

Section 2 describes a laboratory device that simulates a section of a conveyor belt between two adjacent transverse protrusions.

In Section 3, the measured pressure forces acting on the transverse protrusion are presented. The known value of the inclination angle of the folding plate, the gravity of the bulk material, and the shear friction coefficient in the contact area of the material grains with the surface (Plexi or rubber) of the folding plate are analytically calculated.

## 2. Materials and Methods

When transporting bulk materials by belt conveyors of classic construction [45], the disadvantage is the limited angle of inclination of transport δ [deg] (about 18 deg for inward transport and −12 deg for recession transport). The transport slope is defined mainly by the grain size (granulometric composition) of the transported material [46,47] and the coefficient of friction on the contact surfaces of the material grains with the working surface of the conveyor belt.

The grain size of the bulk material is defined by the shape and size of the individual grains [48]. If the grains of material are close to spherical in shape (so-called rotatable grains), the angle of inclination of the transport must be chosen to be significantly lower than the angle of inclination of the transport of non-rotatable grains of material.

**Figure 1:**
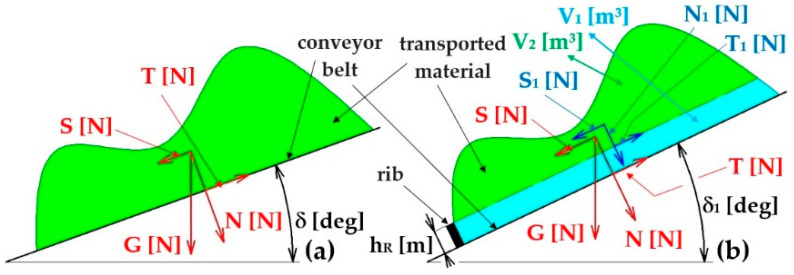
Forces acting on material spread on (**a**) smooth conveyor belt and (**b**) profile conveyor belt with protrusions of height h_R_ [m].

The coefficient of friction f_1_ [−] on the contact surfaces of the material grains with the working surface of the conveyor belt is a dimensionless number defined as the ratio between the friction force (T [N] $(T=N\cdot f_{1}=G\cdot \cos\left(\delta \right)-f_{1}\;\left[N\right]$) and the normal force N [N] ($N=G\cdot \cos\left(\delta \right)\;[N]$). If the sine component of the conveyed material’s gravity S [N] takes on a value higher (see Figure 1a) than the frictional force T [N] of the conveyed material against the conveyor belt, relative movement of the material against the working surface of the conveyor belt occurs.

The coefficient of static friction f_1_ [−], in the contact area of the conveyor belt of the classical construction with the conveyed material, can be expressed according to (1), at the transport inclination δ [deg].

(1) $$f_{1}=\tan\left(\delta \right)\;[-]$$

Assuming they are on the profile conveyor belt [9,10,49], they are installed (perpendicular to the longitudinal axis of the conveyor belt) with protrusions of height h_R_ [m]—see Figure 1b. Additionally, if the magnitude of the friction force T_1_ [N] ($T_{1}=V_{2}-\rho_{s}\cdot g\cdot f_{2}\;\left[N\right]$, where $\rho_{s}\;[\mathrm{kg}\cdot m^{-3}]$ is the bulk density of the material to be conveyed, $f_{2}=\tan\left(\phi \right)\;\left[-\right]\;k$ the coefficient of the internal friction angle, ϕ [deg] the angle of internal friction) is greater than the sine component of the gravity of the volume V_2_ [m^3^] of the conveyed material $S_{1}\;[N]$ ($S_{1}=V_{2}-\rho_{s}\cdot g\cdot \sin(\delta_{1})\;[N]$), the force acting on the protrusion is $F=S_{1}-T_{1}=G\;\cdot \;[\sin\left(\delta_{1}\right)\;-\;f_{1}\cdot \cos(\delta_{1})]\;[N]$.

If the protrusions with regular spacing $t_{R}\;[m]$ are installed in the working branch of the endless loop of the conveyor belt, the total volume V [m^3^] of the conveyed material can be expressed according to Relation (2), if it is taken into account that b=0.9·B−0.05 [m] is the used loading width of the conveyor belt of width B [m] [37] and Θ [deg] is the dynamic spreading angle of the conveyed material.

(2) $$V=A\cdot t_{R}=\frac{1}{6}\cdot b^{2}\cdot \tan\left(\theta \right)\cdot t_{R}\;\left[m^{3}\right]$$

(3) $$A=\int_{\frac{-b}{2}}^{\frac{b}{2}}y_{\left(x\right)}\cdot \mathrm{dx}=\int_{\frac{-b}{2}}^{\frac{b}{2}}\left(h_{\max}-\frac{x^{2}}{-2\cdot p}\right)\cdot \mathrm{dx}\;[m^{2}]$$

(4) $$p=\frac{b}{2\cdot \tan(\Theta )}\;\left[m\right],h_{\max}=\frac{b}{4}\cdot \tan\left(\Theta \right)\;[m]$$

**Figure 2:**
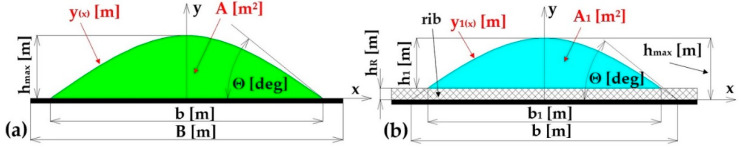
Cross-section of the conveyor belt filling at the used loading width (**a**) b [m], (**b**) b_1_ [m].

The volume of the conveyed material V_2_ [m^3^], see Figure 1b, can be expressed according to Equation (5), assuming the height $h_{1}=h_{\max}-h_{R}\;[m]$ (see Figure 2b).

(5) $$V_{2}=A_{1}\cdot t_{R}=\int_{\frac{-b_{1}}{2}}^{\frac{b_{1}}{2}}\left(h_{1}-\frac{x^{2}}{-2\cdot p}\right)\cdot dx\cdot t_{R}\;\left[m^{3}\right],\;\mathrm{where}\mathrm{is}\;b_{1}=2\cdot \sqrt{2\cdot p\cdot h_{1}}\;[m]$$

**Figure 3:**
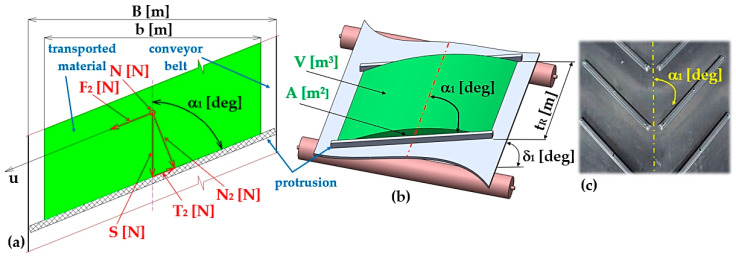
(**a**) Forces acting on a protrusion inclined at an angle $\alpha_{1}$ [deg] with respect to the longitudinal axis of the conveyor belt, (**b**) cross-sectional area A [m^2^] and volume V [m^3^] of the conveyed material between the two protrusions, (**c**) profile conveyor belt [49,50,51].

When the protrusions are inclined by an angle $\alpha_{1}$ [deg] to the longitudinal axis of the profile conveyor belt (of the folding Plexi plate of the laboratory device), see Figure 3a, a normal force $N_{2}=S\cdot \sin(\alpha_{1})\;[N]$ acts on the protrusion and frictional force $T_{2}=S\cdot \cos(\alpha_{1})\cdot f_{1}\;[N]$ which acts in the contact area between the protrusion and the conveyed material. If the magnitude of the force F_2_ [N] is less than the magnitude of the friction force T_2_ [N], there is no relative movement of the conveyed material layer with respect to the working surface of the conveyor belt in the direction of the “u” axis (see Figure 3a).

The equation of motion (6) in the direction of the “u” axis can be expressed according to Figure 3a.

(6) $$F_{2}\leq T_{2}+N\cdot f_{1}\Rightarrow G\cdot \sin\left(\delta_{1}\right)\cdot \cos\left(\alpha_{1}\right)\leq G\cdot \sin\left(\delta_{1}\right)\cdot \sin\left(\alpha_{1}\right)\cdot f_{1}+G\cdot \cos\left(\delta_{1}\right)\cdot f_{1}\;\left[N\right]$$

Equation (6) can be modified to the form (7) provided that $k=f_{1}-\frac{\cos(\delta_{1})}{\sin(\delta_{1})}\;[-]$, $\sin(\alpha_{1})=\frac{2-\tan\left(\frac{\alpha_{1}}{2}\right)}{1+\tan^{2}\left(\frac{\alpha_{1}}{2}\right)}$, $\cos(\alpha_{1})=\frac{1-\tan^{2}\left(\frac{\alpha_{1}}{2}\right)}{1+\tan^{2}\left(\frac{\alpha_{1}}{2}\right)}$.

(7) $$\tan^{2}\left(\frac{\alpha_{1}}{2}\right)\cdot \left(1+k\right)+\tan\left(\frac{\alpha_{1}}{2}\right)\cdot 2\cdot f_{1}+(k-1)=0$$

Solving the quadratic Equation (7), the maximum size of the angle can be found $\alpha_{1}$ [deg] (8), at which the material to be conveyed does not yet start to slide in the direction of the “u” axis on the working surface of the profile conveyor belt.

(8) $$\alpha_{1}\leq 2\cdot \mathrm{atan}\left(\frac{\sqrt{f_{1}^{2}-\left(k^{2}-1\right)}-f_{1}}{k+1}\right)\;[\deg]$$

Table 1 shows the values of the $\alpha_{1}$ [deg] protrusion inclination angle $\delta_{1}\;[\deg]$ (see Figure 3b,c) calculated according to Equation (8) at a transport inclination angle $\delta_{1}\;\left[\deg\right]$ and a shear friction coefficient f_1_ [−].

**Table 1:** Angle of protrusion inclination $\alpha_{1}$ [deg] and coefficient k [−] at the transport inclination angle $\delta_{1}\;[\deg]$ and shear friction coefficient f_1_ = 0.3.

Shear Friction Coefficient f_1_ = 0.3										
δ_1_ [deg]	18	20	22	24	26	28	30	32	34	36
$\alpha_{1}$ [deg] ^1^	11.13	21.16	27.97	33.11	37.20	40.59	43.45	45.92	48.09	50.00
k [−] ^1^	0.924	0.824	0.743	0.674	0.615	0.564	0.52	0.48	0.445	0.413

^1^ see Figure 4.

**Figure 4:**
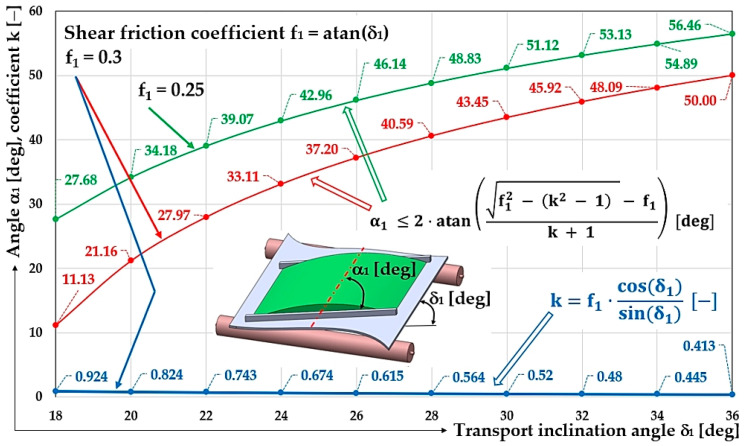
Angle of protrusion inclination $\alpha_{1}$ [deg] and coefficient k [−] at the transport inclination angle $\delta_{1}$ [deg] and a shear friction coefficient f_1_ [−].

Table 2 shows the values of the $\alpha_{1}$ [deg] protrusion inclination angle $\delta_{1}\;[\deg]$ (see Figure 3b,c) calculated according to Equation (8) at a transport inclination angle $\delta_{1}\;\left[\deg\right]$ and a shear friction coefficient f_1_ [−].

**Figure 5:**
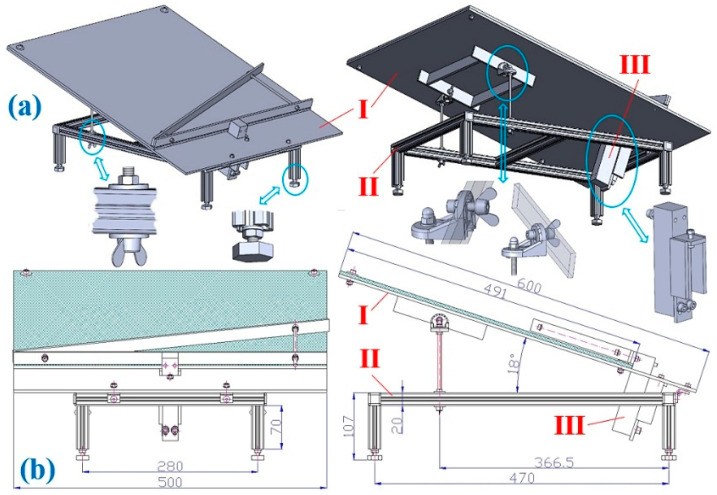
Structural design of the laboratory device for measuring the compressive force acting on the protrusion: (**a**) 3D model created in SolidWorks 2024, (**b**) dimensional sketch.

In order to be able to obtain accurate values of the applied compressive force $F_{\left(\delta_{1},\alpha \right)k}\;[N]$ on the transverse protrusion inclined to the longitudinal axis of the folding plate by an angle $\alpha_{1}$ [deg], a laboratory device was designed and constructed, see Figure 5, which consists of three basic parts, I—the folding plate (inclination angle with respect to the horizontal $\delta_{1}=18\;\deg÷38\;\deg$ plane), II—the supporting frame, and III—the strain gauge load sensor PW2G C3—12 kg (Hottinger Brüel & Kjaer Gmb, Darmstadt, Germany) [52], detecting the magnitude of the compressive forces $F_{\mathrm{FM}\left(\delta_{1}\right),k}\;[N]$ and $F_{M\left(\delta_{1}\right),k}\;[N]$.

**Figure 6:**
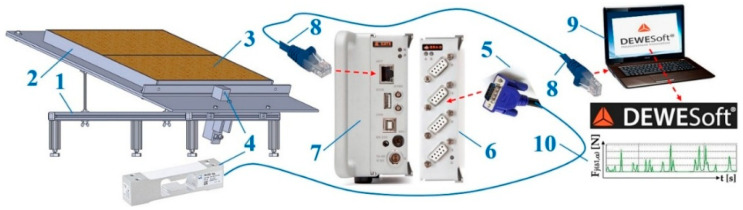
Measuring chain using DS NET to sense tensile force $F_{M\left(\delta_{1}\right),k}\;[N]$. 1—laboratory device, 2—Plexi frame, 3—bulk material, 4—load sensor [52], 5—D-Sub plug, 6—BR4—D measuring module [11], 7—gateway module DS GATE [53], 8—network cable, 9—laptop, 10—DEWESoft X2 SP5 software (version number SP5) [54].

On the inclined surface (at a predetermined angle of inclination to the horizontal $\delta_{1}=18\;\deg÷38\;\deg$) of the laboratory equipment (1), see Figure 6, a Plexi frame was placed (2) of gravity $G_{R}$ = 3.26 N (weight $m_{R}$ = 0.332 kg), into which the bulk material was placed (3) of known gravity G [N]. The applied compressive force on the transverse protrusion from the Plexi frame $F_{\mathrm{FM}\left(\delta_{1}\right),k}\;[N]$ and also from the gravity component of the bulk material (river gravel, grain size 4 ÷ 8 mm) $F_{M\left(\delta_{1}\right),k}\;[N]$ were detected by a load sensor (4) [52] at a known value of the inclination angle $\delta_{1}$ [deg] of the folding plate.

To determine the coefficient of internal friction angle of the bulk material $f_{2}$ [−], laboratory tests were carried out as follows. The first plastic frame was placed on the working surface (Plexi or rubber) of the folding plate, which was slowly deflected by an angle $\delta_{1}$ [deg] to the horizontal plane. The inner space of this plastic frame was completely filled with loose material of known gravity G [N].

A second plastic frame was placed on top of the first plastic frame and a batch of bulk material of known gravity $G_{1}$ [N] was slowly poured into it.

The lower plastic frame met the transverse protrusion with its front surface. In the contact area (corresponding to the plane parallel to the surface of the folding plate of the laboratory equipment, spaced by the height of the lower frame $h_{R}$ [m]), the material grains of the bulk material placed in the lower frame came into contact with the material grains placed in the upper frame.

If the gravity component of the bulk material of charge $G_{1}\cdot \sin\left(\delta_{1k}\right)$ [N] has taken on a magnitude greater than the frictional force $G_{1}\cdot \cos\left(\delta_{1k}\right)-f_{2}$ [N] (acting in the contact area of the shear plane), relative movement of the upper frame relative to the lower frame has occurred. The angle $\delta_{1k}$ [deg] is considered to be the angle of internal friction of the bulk material (river gravel, rounded grain edges). By substituting the value $\delta_{1k}$ [deg] into Equation (1), the coefficient of the internal friction angle of the bulk material $f_{2}$ [−] can be analytically calculated.

A load sensor cable equipped with a D-Sub plug (5) was plugged into the socket of the measuring module BR4-D 6 [53] of the strain gauge apparatus DS NET during the laboratory measurements. A PC 9 (ASUS K72JR—TY131 laptop (ALZA.CZ, Ostrava, Czech Republic)) was connected to the DS NET strain gauge (7) using a network cable with RJ—45 connectors (8) at both ends.

## 3. Results

Whether it is necessary to measure pressure forces acting on conveyor belt protrusions depends on the specific application, the conveyor design, and the operating conditions. In many cases, such measurements are beneficial, although not always absolutely essential.

Experimental analysis is appropriate when the protrusions mounted on the conveyor belt are subjected to high loads (e.g., steep conveyors, heavy or abrasive materials). It is also advisable when designing new cleat geometries or developing new rubber compounds for cleats and conveyor belt cover layers.

The experimental analysis (described in this article) was carried out to verify the accuracy of the computational models. The objective was to validate whether the recommended inclination angle (in the range of 18 deg to 40 deg) for conveying bulk material (river gravel) using a conveyor belt with transverse cleats is suitable for operation with this type of conveyor belt in a belt conveyor system.

From the values detected by the load sensor [52] $F_{M\left(\delta_{1}\right),k}\;[N]$ in the DEWESoft X software environment [54], with the known value of the material gravity G [N] and the inclination angle of the folding plate $\delta_{1}$ [deg], the friction coefficient $f_{1j\left(\delta_{1},\alpha \right),k}\;\left[-\right]$ was calculated according to Equation (9), where “i” defines the surface of the folding plate i = P for Plexi, i = R for rubber.

(9) $$f_{1i\left(\delta_{1},\alpha \right),k}=\frac{G\cdot \sin\left(\delta_{1}\right)-F_{\left(\delta_{1},\alpha \right),k}}{\cos\left(\delta_{1}\right)}\;\left[-\right]$$

### 3.1. Plexi Contact Surface, Inclination of Protrusion $\alpha_{1}=90\;deg$

Table 3 presents the measured values of the pressure forces $F_{0M\left(\delta_{1}\right),k}\;[N]$, $F_{\mathrm{FM}\left(\delta_{1}\right),k}\;[N]$ and $F_{M\left(\delta_{1}\right),k}\;[N]$ acting on the protrusion at the angles of inclination of the folding plate $\delta_{1}=18\;\deg$ and $\delta_{1}=23\;\deg$.

The quantity $F_{0M\left(\delta_{1}\right),k}\;[N]$ expresses the force detected by the load sensor [52] at the moment of the start of the measurement. The non-zero magnitude of the compressive force $F_{0M\left(\delta_{1}\right),k}\;[N]$ is due to the fact that the load sensor [52] was calibrated (with a calibrated 2 kg weight load) in the horizontal position, without the fasteners used and without the plastic plate (500 mm long, 20 mm high and 5 mm thick) that simulates the transverse protrusion.

The pressure force $F_{\mathrm{FM}\left(\delta_{1}\right),k}\;[N]$ defines the force generated by a Plexi frame (weight 0.332 kg, gravity 3.26 N) placed on the surface of the folding plate on the load sensor [52].

Loose material (river gravel of 4 ÷ 8 mm grain size) of known gravity is poured into the inner space of the Plexi frame G [N]. In the DEWESoft software environment, the magnitude of the force $F_{M\left(\delta_{1}\right),k}\;[N]$, acting on the load sensor [52], from the gravity component G [N] of the bulk material is recorded.

From the measured values of the forces $F_{0M\left(\delta_{1}\right),k}\;[N]$, $F_{\mathrm{FM}\left(\delta_{1}\right),k}\;[N]$ and $F_{M\left(\delta_{1}\right),k}\;[N]$ and the known value of the gravity G [N] of the bulk material, the actual magnitude of the compressive force $F_{\left(\delta_{1},\alpha \right),k}\;\left[N\right]$ acting on the protrusion from the gravity component of the bulk material was calculated. From this force $F_{\left(\delta_{1,\alpha }\right),k}\;\left[N\right]$, the gravity of the bulk material G [N] and the angle of inclination of the folding Plexi plate, the shear friction coefficient $f_{1j\left(\delta_{1},\alpha \right),k}\;\left[-\right]$ was calculated according to Equation (8).

From three (n = 3) repeated measurements under the same technical conditions, the arithmetic mean $f_{1j\left(\delta_{1},\alpha \right)\mathrm{AM}}\;\left[-\right]$ and the marginal error $\kappa_{\left(\beta ,n\right)\delta 1}\;\left[-\right]$ (10) were calculated according to Student’s distribution [55].

(10) $$\kappa_{\left(\beta ,n\right)\delta_{1}}=t_{\beta ,n}\cdot \bar{s}\;\left[-\right],$$

where $t_{\beta ,n}\;\left[-\right]$ is the Student’s coefficient (for the chosen risk β = 5%) and the number of measured values n=3 can be determined according to [55] $t_{\beta ,n}=t_{5\%,3}=4.3$); $\bar{s}\;[-]$ is the standard deviation of the arithmetic mean (11).

(11) $$\bar{s}\approx \frac{5}{4}\cdot \frac{\sum_{k=1}^{3}\left|\Delta_{k}\right|}{n\cdot \sqrt{n-1}}=\frac{5}{4}\cdot \frac{\sum_{k=1}^{3}|f_{ij\left(\delta 1,\alpha \right),k}\;-f_{ij(\delta 1,\alpha )\mathrm{AM}}|}{n\cdot \sqrt{n-1}}\;[-]$$

**Figure 7:**
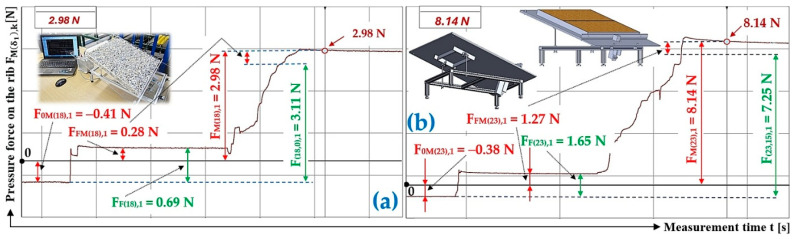
Measured value of pressure force $F_{M\left(\delta_{1}\right),k}\;[N]$ in the DeweSoft software environment for a protrusion angle of $\alpha_{1}=90\;\deg$ and a conveyor belt inclination angle of (**a**) $\delta_{1}$ = 18 deg, (**b**) $\delta_{1}$ = 23 deg.

Figure 7a presents the time history of the compressive force measured by the load sensor [52], generated by a bulk material of known gravity G = 70 N, distributed on the surface of a folding Plexi plate inclined to the horizontal plane by an angle δ1 = 18 deg. The Plexi plate (simulating the protrusion) is inclined at an angle $\alpha_{1}$ = 90 deg to the longitudinal axis of the folding Plexi plate (width b = 400 mm).

The time history of the compressive force (a batch of bulk material with a gravity of G = 70 N) detected by the load sensor [52] at an angle of inclination of the Plexi plate $\delta_{1}$ = 23 deg is indicated in Figure 7b.

The magnitude of three times the measured compressive force of the bulk material of gravity G = 70 N acting on the protrusion (located at an angle $\alpha_{1}$= 90 deg to the longitudinal axis) of the folding plate, deflected by an angle $\delta_{1}=28\;\deg$ and $\delta_{1}=32\;\deg$ from the horizontal plane, is given in Table 4.

**Figure 8:**
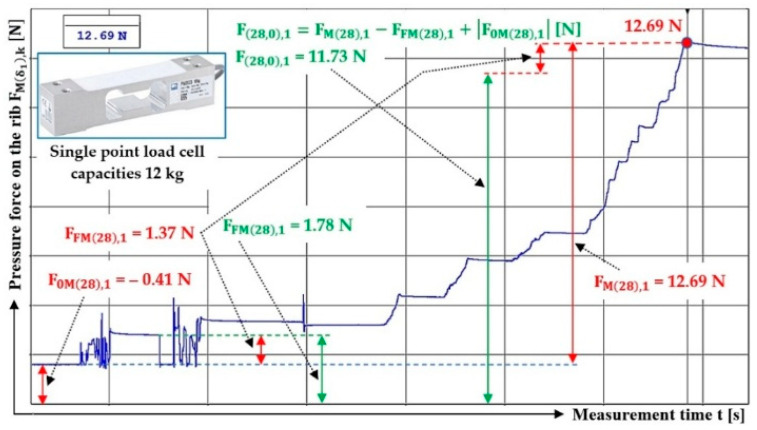
Measured value of pressure force $F_{M\left(\delta_{1}\right),k}\;[N]$ in the DeweSoft software environment for the angle of protrusion $\alpha_{1}=90\;\deg$ and the angle of inclination of the conveyor belt $\delta_{1}=28\;\deg$.

The time history of the compressive force (a batch of bulk material with a gravity of G = 70 N) detected by the load sensor [52] at a Plexi plate inclination angle (simulating a conveyor belt) $\delta_{1}$ = 23 deg and a lug inclination angle $\alpha_{1}$ = 90 deg with respect to the longitudinal axis of the folding plate is indicated in Figure 8.

The values measured by the load sensor, $F_{0M\left(38\right),k}\;[N]$, $F_{\mathrm{FM}\left(38\right),k}\;[N]$, and $F_{M\left(98\right),k}\;[N]$ [52], acting on the Plexi plate (simulating the transverse protrusion) of the laboratory device (see Figure 5) are (for the inclination angle of the folding plate $\delta_{1}=38\;\deg$ and the inclination angle of the protrusion α=0 deg) shown in Table 5.

**Figure 9:**
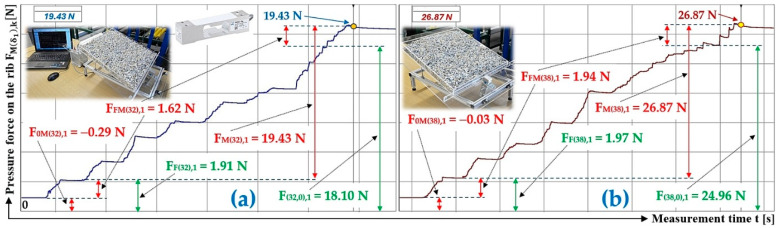
Measured value of pressure force $F_{M\left(\delta_{1}\right),k}\;[N]$ in the DeweSoft software environment for a protrusion angle of $\alpha_{1}=90\;\deg$ and a conveyor belt inclination angle of (**a**) $\delta_{1}$ = 32 deg, (**b**) $\delta_{1}$ = 38 deg.

Figure 9a presents the time history of the measured compressive force of a bulk material of gravity G = 70 N, spread on the surface of a folding Plexi plate inclined to the horizontal plane at an angle $\delta_{1}$ = 32 deg.

The time history of the compressive force (a batch of bulk material with a gravity of G = 70 N) detected by the load sensor [52] at an angle of inclination of the Plexi plate $\delta_{1}$ = 38 deg is indicated in Figure 9b.

### 3.2. Plexi Contact Surface, Inclination of Protrusion $\alpha_{1}=75\;deg$

The measurement of the compressive force of the bulk material of gravity G [N] acting on a transverse Plexi plate of height 20 mm (simulating a transverse protrusion), placed at an angle $\alpha_{1}=75\;\deg$ to the longitudinal axis of the folding plate (simulating a conveyor belt) by a precision instrument (strain gauge load sensor PW2G C3—12 kg) and carefully repeated under the same conditions does not yield the same values. However, the measurand has one actual (accurate) value for the given measurement conditions.

Table 6 shows the measured values of the compressive forces $F_{0M\left(\delta_{1}\right),k}\;[N]$, $F_{\mathrm{FM}\left(\delta_{1}\right),k}\;[N]$, and $F_{M\left(\delta_{1}\right),k}\;[N]$ (of bulk material of gravity G = 45 N acting on the Plexi plate, mechanically attached to the load sensor [52] at an angle $\alpha_{1}=75\;\deg$ with respect to the transverse axis of the folding Plexi plate) for three repeated measurements for the inclination angle of the folding plate $\delta_{1}$ = 18 deg and $\delta_{1}$ = 23 deg. From these measured forces, the arithmetic mean $f_{1j\left(\delta_{1},\alpha \right),\mathrm{AM}}\;\left[-\right]$ and the extreme error $\kappa_{\left(\beta ,n\right)\delta 1}\;[-]$ are calculated according to Equation (9).

**Figure 10:**
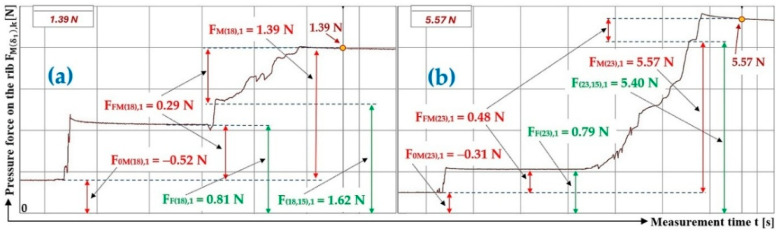
Measured values in the DeweSoft software environment for $\alpha_{1}=75\;\deg$ and the angle of inclination of the conveyor belt (**a**) $\delta_{1}$ = 18 deg, (**b**) $\delta_{1}$ = 23 deg.

Figure 10a presents the time history of the compressive force measured by the load sensor [52], generated by a bulk material of known gravity G = 45 N, distributed on the surface of a folding Plexi plate inclined to the horizontal plane by an angle $\delta_{1}$ = 18 deg. The Plexi plate (simulating the protrusion) is inclined at an angle $\alpha_{1}=75\;\deg$ to the longitudinal axis of the folding Plexi plate.

The time history of the compressive force (a batch of bulk material with a gravity of G = 45 N) detected by the load sensor [52] at an angle of inclination of the Plexi plate $\delta_{1}$ = 23 deg is indicated in Figure 10b.

The magnitude of three times the measured compressive force of the bulk material of gravity G = 70 N acting on the protrusion located at an angle $\alpha_{1}=75\;\deg$ to the longitudinal axis of the folding plate, deflected by an angle $\delta_{1}$ = 28 deg and $\delta_{1}$ = 32 deg from the horizontal plane, is given in Table 7.

**Figure 11:**
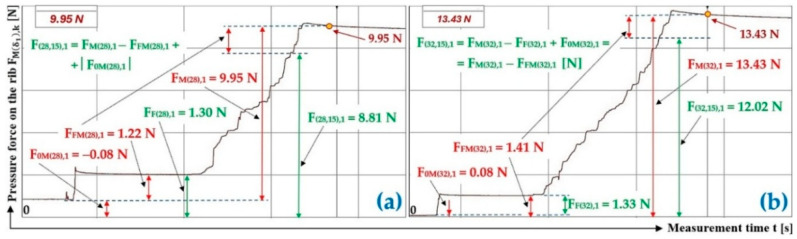
Measured values in the DeweSoft software environment for $\alpha_{1}=75\;\deg$ and the angle of inclination of the conveyor belt (**a**) $\delta_{1}$ = 28 deg, (**b**) $\delta_{1}$ = 32 deg.

Figure 11a presents the time history of the compressive force measured by the load sensor [52], generated by a bulk material of known gravity G = 45 N, distributed on the surface of a folding Plexi plate inclined to the horizontal plane by an angle $\delta_{1}$ = 28 deg. The Plexi plate (simulating the protrusion) is inclined at an angle $\alpha_{1}=75\;\deg$ to the longitudinal axis of the folding Plexi plate.

The time history of the compressive force (a batch of bulk material with a gravity of G = 45 N) detected by the load sensor [52] at an angle of inclination of the Plexi plate $\delta_{1}$ = 32 deg is indicated in Figure 11b.

The values measured by the load sensor, $F_{0M\left(38\right),k}\;[N]$, $F_{\mathrm{FM}\left(38\right),k}\;[N]$, and $F_{M\left(98\right),k}\;[N]$ [52], acting on the Plexi plate (simulating the transverse protrusion) of the laboratory device (see Figure 5) are (for the inclination angle of the folding plate $\delta_{1}=38\;\deg$ and the inclination angle of the protrusion $\alpha_{1}=75\;\deg$) shown in Table 8.

**Figure 12:**
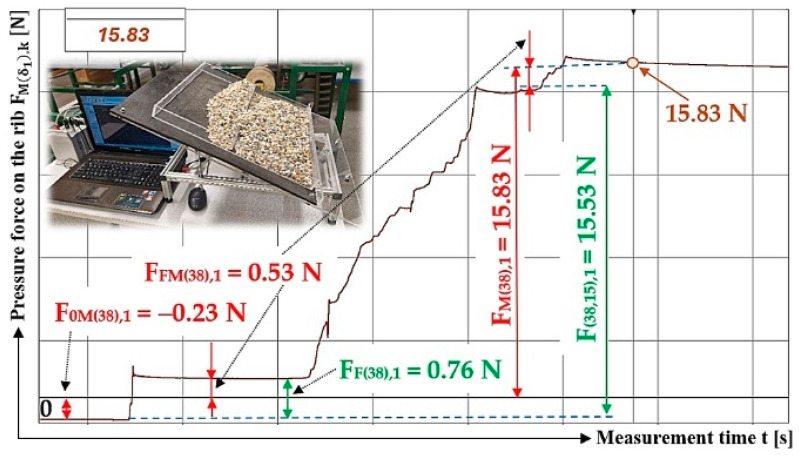
Measured values in the DeweSoft software environment for $\alpha_{1}=75\;\deg$ and the angle of inclination of the conveyor belt $\delta_{1}$ = 38 deg.

The time history of the compressive force (a batch of bulk material with a gravity of G = 45 N) detected by the load sensor [52] at a Plexi plate inclination angle (simulating a conveyor belt) $\delta_{1}$ = 38 deg and a lug inclination angle $\alpha_{1}=75\;\deg$ with respect to the longitudinal axis of the folding plate, is indicated in Figure 12.

### 3.3. Rubber Contact Surface, Inclination of Protrusion $\alpha_{1}=90\;deg$

Table 9 shows the measured values of the compressive forces $F_{0M\left(\delta_{1}\right),k}\;[N]$, $\;F_{\mathrm{FM}\left(\delta_{1}\right),k}\;[N]$, ${\mathrm{and}\;F}_{M\left(\delta_{1}\right),k}\;[N]$ (of bulk material of gravity G = 70 N (G = 41 N) acting on the Plexi plate, mechanically attached to the load sensor [52] at an angle $\alpha_{1}=90\;\deg$ with respect to the longitudinal axis of the folding Plexi plate) of three repeated measurements for the inclination angle of the folding plate $\delta_{1}$ = 18 deg ($\delta_{1}$ = 23 deg). From these measured forces, the arithmetic mean $f_{1j\left(\delta_{1},\alpha \right),\mathrm{AM}}\;\left[-\right]$ and the extreme error $\kappa_{\left(\beta ,n\right)\delta 1}\;[-]$ are calculated according to Equation (9).

**Figure 13:**
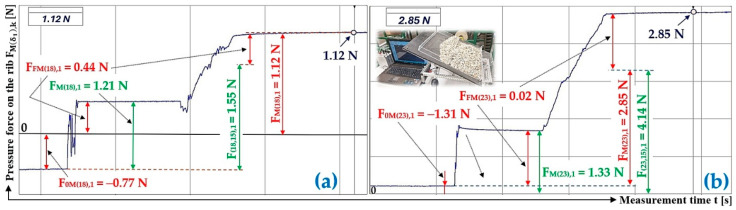
Measured values in the DeweSoft software environment for $\alpha_{1}=90\;\deg$ and the angle of inclination of the conveyor belt (**a**) $\delta_{1}$ = 18 deg, (**b**) $\delta_{1}$ = 23 deg.

Figure 13a presents the time history of the compressive force measured by the load sensor [52], generated by a bulk material of known gravity G = 70 N, distributed on the surface of a folding Plexi plate inclined to the horizontal plane by an angle $\delta_{1}$ = 18 deg. The Plexi plate (simulating the protrusion) is inclined at an angle α = 0 deg to the transverse axis of the folding Plexi plate.

The time history of the compressive force (a batch of bulk material with a gravity of G = 41 N) detected by the load sensor [52] at an angle of inclination of the Plexi plate $\delta_{1}$ = 23 deg is indicated in Figure 13b.

The magnitude of three times the measured compressive force of the bulk material of gravity G = 41 N acting on the protrusion (located at an angle $\alpha_{1}=90\;\deg$ to the longitudinal axis) of the folding plate, deflected by an angle $\delta_{1}$ = 28 deg and $\delta_{1}$ = 32 deg from the horizontal plane, is given in Table 10.

**Figure 14:**
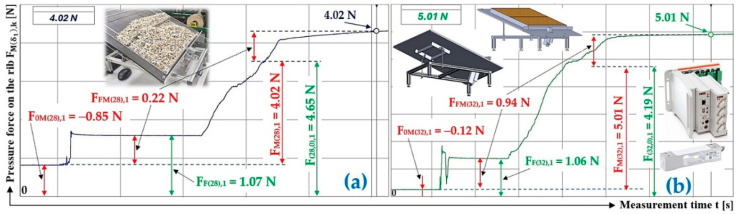
Measured values in the DeweSoft software environment for $\alpha_{1}=90\;\deg$ and the angle of inclination of the conveyor belt (**a**) $\delta_{1}$ = 28 deg, (**b**) $\delta_{1}$ = 32 deg.

Figure 14a presents the time history of the compressive force measured by the load sensor [52], generated by a bulk material of known gravity G = 41 N, distributed on the surface of a folding Plexi plate inclined to the horizontal plane by an angle $\delta_{1}$ = 28 deg. The Plexi plate (simulating the protrusion) is inclined at an angle $\alpha_{1}=90\;\deg$ to the longitudinal axis of the folding Plexi plate.

The time history of the compressive force (a batch of bulk material with a gravity of G = 41 N) detected by the load sensor [52] at an angle of inclination of the Plexi plate $\delta_{1}$ = 32 deg is indicated in Figure 14b.

The values measured by the load sensor, $F_{0M\left(38\right),k}\;[N]$, $F_{\mathrm{FM}\left(38\right),k}\;[N]$, and $F_{M\left(98\right),k}\;[N]$ [52], acting on the Plexi plate (simulating the transverse protrusion) of the laboratory device (see Figure 5) are (for the inclination angle of the folding plate $\delta_{1}=38\;\deg$ and the inclination angle of the protrusion $\alpha_{1}=90\;\deg$) shown in Table 11.

**Figure 15:**
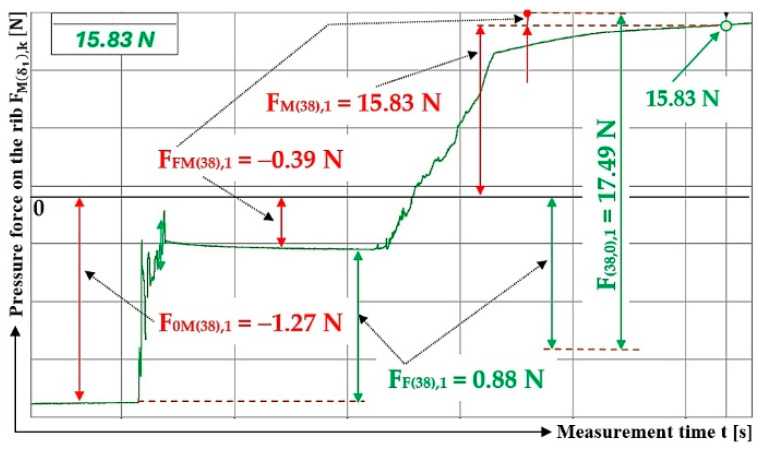
Measured values in the DeweSoft software environment for $\alpha_{1}=90\;\deg$ and the angle of inclination of the conveyor belt $\delta_{1}$ = 38 deg.

The time history of the compressive force (a batch of bulk material with a gravity of G = 70 N) detected by the load sensor [52] at a Plexi plate inclination angle (simulating a conveyor belt) $\delta_{1}$ = 38 deg and a lug inclination angle $\alpha_{1}=90\;\deg$ with respect to the transverse axis of the folding plate is indicated in Figure 15.

### 3.4. Rubber Contact Surface, Inclination of Protrusion $\alpha_{1}=75\;deg$

Table 12 shows the measured values of the compressive forces $F_{0M\left(\delta_{1}\right),k}\;[N]$,$\;F_{\mathrm{FM}\left(\delta_{1}\right),k}\;[N]$, and $F_{M\left(\delta_{1}\right),k}\;[N]$ (of bulk material of gravity G = 41 N) acting on the Plexi plate (which is mechanically attached to the load sensor [52] at an angle $\alpha_{1}=75\;\deg$ with respect to the longitudinal axis of the folding Plexi plate) of three repeated measurements for the inclination angle of the folding plate $\delta_{1}$ = 23 deg ($\delta_{1}$ = 28 deg). From these measured forces, the arithmetic mean $f_{1j\left(\delta_{1},\alpha \right),\mathrm{AM}}\;\left[-\right]$ and the extreme error $\kappa_{(\beta ,n)\delta 1}\;[-]$ are calculated according to Equation (9).

**Figure 16:**
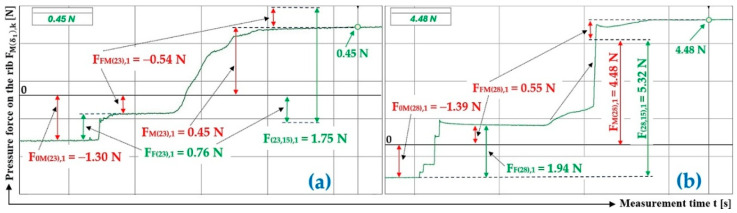
Measured values in the DeweSoft software environment for $\alpha_{1}=75\;\deg$ and the angle of inclination of the conveyor belt (**a**) $\delta_{1}$ = 23 deg, (**b**) $\delta_{1}$ = 28 deg.

Figure 16a presents the time history of the compressive force measured by the load sensor [52], generated by a bulk material of known gravity G = 41 N, distributed on the surface of a folding Plexi plate inclined to the horizontal plane by an angle $\delta_{1}$ = 23 deg. The Plexi plate (simulating the protrusion) is inclined at an angle $\alpha_{1}=75\;\deg$ to the longitudinal axis of the folding Plexi plate.

The time history of the compressive force (a batch of bulk material with a gravity of G = 41 N) detected by the load sensor [52] at an angle of inclination of the Plexi plate $\delta_{1}$ = 28 deg is indicated in Figure 16b.

The magnitude of three times the measured compressive force of the bulk material of gravity G = 41 N acting on the protrusion (located at an angle $\alpha_{1}=75\;\deg$ to the longitudinal axis) of the folding plate, deflected by an angle $\delta_{1}$ = 32 deg and $\delta_{1}$ = 38 deg from the horizontal plane, is given in Table 13.

**Figure 17:**
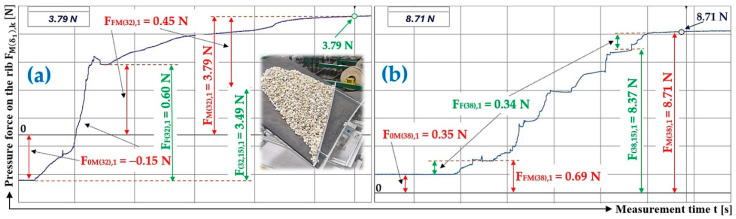
Measured values in the DeweSoft software environment for $\alpha_{1}=75\;\deg$ and the angle of inclination of the conveyor belt (**a**) $\delta_{1}$ = 32 deg, (**b**) $\delta_{1}$ = 38 deg.

Figure 17a presents the time history of the compressive force measured by the load sensor [52], generated by a bulk material of known gravity G = 41 N, distributed on the surface of a folding Plexi plate inclined to the horizontal plane by an angle $\delta_{1}$ = 32 deg. The Plexi plate (simulating the protrusion) is inclined at an angle α=75 deg to the longitudinal axis of the folding Plexi plate.

The time history of the compressive force (a batch of bulk material with a gravity of G = 41 N) detected by the load sensor [52] at an angle of inclination of the Plexi plate $\delta_{1}$ = 88 deg is indicated in Figure 17b.

Table 14 shows the mean values $f_{1j\left(\delta_{1},\alpha \right),\mathrm{AM}}\;\left[-\right]$, calculated according to Equation (8), of the shear friction coefficients $f_{1j\left(\delta_{1},\alpha \right),k}\;\left[-\right]$ of bulk material (river gravel of grain size 4 ÷ 8 mm) on the contact surface of the folding plate, inclined at an angle $\delta_{1}$ [deg].

**Figure 18:**
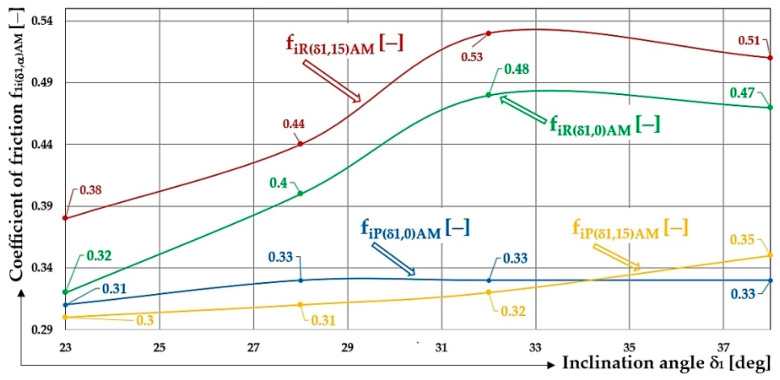
Values of shear friction coefficient $f_{1j\left(\delta_{1},\alpha \right),k}\;\left[-\right]$ of bulk material (river gravel of 4 ÷ 8 mm grain size) in the contact area of the folding plate, inclined at an angle $\delta_{1}$ [deg].

Figure 18 shows the calculated values, according to Equation (8), of the shear friction coefficient $f_{1j\left(\delta_{1},\alpha \right),k}\;\left[-\right]$ of the bulk material (river gravel of 4 ÷ 8 mm grain size) in the contact area (j = P—Plexi, j = R—rubber) of the folding plate inclined at an angle $\delta_{1}$ [deg].

### 3.5. Determination of the Coefficient of Internal Friction of Bulk Material

**Figure 19:**
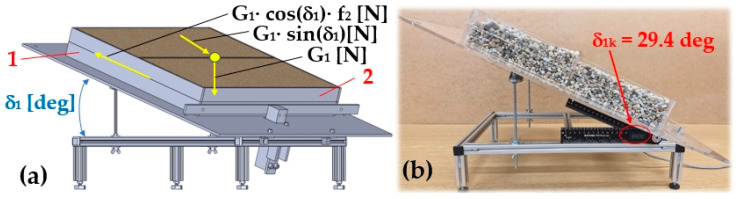
Measurement of the inclination angle $\delta_{1k}$ [deg]. (**a**) Applied forces during the inclination change in the folding plate, (**b**) realized measurement. 1—lower frame, 2—upper frame.

Figure 19a shows the method of measurement on the laboratory equipment to determine the angle of inclination $\delta_{1k}$ [deg] at which the movement of a volume of a batch of bulk material spread inside the upper frame will occur (2), relative to the bulk material inside the lower frame (1).

Figure 19b presents the measured inclination angle $\delta_{1k}$ = 29.4 deg of the folding plate of the laboratory device, at which the movement of the bulk batch of bulk material spread inside the upper frame relative to the bulk material placed in the lower frame began.

Table 15 shows the measured values of the inclination angle $\delta_{1k}$ [deg] of the folding plate of the laboratory device at which the relative motion of the upper frame (1), see Figure 19a, to the lower frame (2) began. From these five (n = 5) repeated measurements of the inclination angle $\delta_{1k}$ [deg] under the same technical conditions, the magnitudes of the internal friction angle coefficient $f_{2,k}$ [−] were calculated according to Equation (1) (for the number of measurements k = 1 to n). From the calculated magnitudes of the coefficients of the internal friction angle $f_{2,k}$ [−], the arithmetic mean $f_{2,\mathrm{AM}}$ [−] and the extreme error $\kappa_{\left(\beta ,n\right)}\;[-]$ were calculated according to Equation (9). For the chosen risk β = 5% and the number of measured values n=5, the Student’s coefficient $t_{\beta ,n}=t_{5\%,5}=2.78$ can be determined according to [55].

## 4. Discussion

From the values obtained by measuring the pressure forces acting on a transverse protrusion inclined by an angle $\alpha_{1}$ [deg] with respect to the longitudinal (α [deg] transverse) axis of the folding Plexi plate (simulating a section of the conveyor belt between two adjacent protrusions), it follows that at low angles of inclination $\delta_{1}$ [deg] of the folding Plexi plate, the magnitude of the coefficient takes on a lower value than in the case of higher angles of inclination $\delta_{1}$ [deg] of the folding Plexi plate. This fact can be expressed by the fact that the friction force $T=G\cdot \cos\left(\delta_{1}\right)-f_{1j\left(\delta_{1},\alpha \right)\mathrm{AM}}\;[N]$ in the contact area of the conveyed material grains with the folding Plexi plate takes a value higher than the sine component of the gravity $S=G\cdot \sin\left(\delta_{1}\right)\;[N]$ of the conveyed material. The friction force T [N] prevents the relative (backward) movement of the material against the direction of movement of the conveyor belt. At low angles of inclination $\delta_{1}$ [deg] of the conveyor belt, there is no backward movement of the conveyed material even if no transverse protrusion is installed on the conveyor belt.

If a bulk material of gravity G [N] is in contact with the Plexi (j = P) surface of the folding plate (simulating the working surface of the conveyor belt) at an angle of inclination of the protrusion α = 0 deg and an angle of the folding plate $\delta_{1}=18\;\deg$ ($\delta_{1}=23\;\deg$), the friction coefficient takes the value $f_{1P\left(18.0\right)\mathrm{AM}}=0.28$ ($f_{1P\left(23.0\right)\mathrm{AM}}=0.30$). Both these friction coefficient values are not the actual value of the friction coefficient of the bulk material (river gravel) against the Plexi surface of the folding plate. The actual magnitude of the friction coefficient $f_{1P\left(28.0\right)\mathrm{AM}}=0.33$ corresponds to the condition where the friction force T [N] on the contact surface of the material grains conveyed with the Plexi surface of the folding plate (inclined to the horizontal plane by an angle $\delta_{1}\geq 28\;\deg$), takes a value lower than the sine component of the gravity S [N] of the material conveyed.

If the bulk material is placed on the rubber (j = R) surface of the folding plate, then, when the angle of inclination of the protrusion α = 0 deg and the inclination of the folding plate $\delta_{1}=23\;\deg$, ${(\delta }_{1}=28\;\deg$), the friction coefficient takes the values $f_{1R\left(18.0\right)\mathrm{AM}}=0.38$ ($f_{1R\left(23.0\right)\mathrm{AM}}=0.32$ and $f_{1R\left(28.0\right)\mathrm{AM}}=0.40$). These three values of the coefficient of friction are not the actual values of the coefficient of friction of the loose material (river gravel) against the rubber surface of the folding plate. The actual magnitude of the coefficient of friction is $f_{1R\left(32.0\right)\mathrm{AM}}=0.48,$ which corresponds to the condition where the friction force T [N] on the contact surface of the material grains conveyed with the rubber surface of the folding plate (inclined to the horizontal plane by an angle $\delta_{1}\geq 32\;\deg$), takes a value lower than the sine component of the gravity S [N] of the material conveyed.

In the case that a bulk material of gravity G [N] is placed on the Plexi surface of the folding plate, the friction coefficient takes the value $f_{1P\left(18.15\right)\mathrm{AM}}=0.28$ ($f_{1P\left(23.15\right)\mathrm{AM}}=0.30$ and $f_{1P\left(28.15\right)\mathrm{AM}}=0.31$) when the angle of inclination of the protrusion α = 15 deg and the angle of inclination of the folding plate $\delta_{1}=18\;\deg$, ${(\delta }_{1}=23\;\deg$ and $\delta_{1}=28\;\deg$);. These three friction coefficient values are again not the actual value of the friction coefficient of the bulk material (river gravel) against the plastic surface of the folding plate. The actual magnitude of the friction coefficient is $f_{1P\left(32.15\right)\mathrm{AM}}=0.34$.

If the bulk material is placed on the rubber (j = R) surface of the folding plate, then, with the angle of inclination of the protrusion α = 15 deg and the inclination of the folding plate $\delta_{1}=23\;\deg$, ${(\delta }_{1}=28\;\deg$), the friction coefficient takes the value $f_{1R\left(23.15\right)\mathrm{AM}}=0.38$ ($f_{1R\left(28.15\right)\mathrm{AM}}=0.40$). These two values of the coefficient of friction are not the actual values of the coefficient of friction of the bulk material (river gravel) against the rubber surface of the folding plate. The actual magnitude of the coefficient of friction is $f_{1R\left(32.15\right)\mathrm{AM}}=0.52,$ which corresponds to the condition where the friction force T [N] on the contact surface of the material grains conveyed with the rubber surface of the folding plate (inclined to the horizontal plane by an angle $\delta_{1}\geq 32\;\deg$) takes a value lower than the sine component of the gravity S [N] of the material conveyed [56].

The static coefficient of the internal friction angle of a bulk material [46] is a basic mechanical characteristic of bulk materials that describes the resistance of a material to shear between its particles at rest [47,48]. For the loose material river gravel (dry, rounded grains) the coefficient of the internal friction angle takes the value $f_{2}$ = 0.45 ÷ 0.65 [57,58]. The measurements carried out on the laboratory equipment, see Section 3.5, show that the mean value of the static coefficient of the internal friction angle of the bulk material—dry river gravel—takes the value $f_{2,\mathrm{AM}}$ = 0.54.

The internal friction angle of a bulk material is influenced by a number of factors related to the properties of the particles and their arrangement. The granulometry (shape and grain size) of the bulk material, moisture, compaction, and the method of loading have a major influence on the magnitude of the internal friction angle. Given the operating conditions characterized by dynamic loads and vibrations under which profile conveyor belts are commonly used, the limiting angle of inclination of the conveyor, at which the grains of the conveyed material move against the direction of movement of the conveyor belt, can be expected to be lower than the angle $\delta_{2}=\mathrm{atan}\left(f_{2,\mathrm{AM}}\right)=\mathrm{atan}\left(0.54\right)\approx$ 28 deg. The difference between the static and dynamic angle of internal friction, resulting from the state of particle motion and the magnitude of frictional resistance of the bulk material conveyed by the profile belt, must be taken into account when determining the operating conditions for a particular type of material and its mechanical–physical properties.

Laboratory measurements of the forces acting on conveyor belt protrusions using the measuring device presented in this article contribute to real belt conveyor applications in the following areas:

− Knowledge of the actual forces acting on protrusions located on the carrying side of the profiled conveyor belt enables designers to properly dimension their geometry, material properties, and attachment method.
− Force measurements provide input data on the operational loading of cleats in profiled conveyor belts used in practice. The accurate determination of the actual protrusion loads allows for the adjustment of operating parameters (belt speed, inclination angle, material throughput), thereby reducing the risk of fatigue damage and extending the service life of the belt conveyor.
− Understanding the real force conditions also makes it possible to optimize friction and resistance during material transport, which can lead to the reduced energy consumption of the entire conveying system.

## 5. Conclusions

At present, there is no clear way to determine the cross-section of the belt fill or to determine theoretically the quantity of bulk material that can be moved per unit time by profile conveyor belts inclined at a certain angle to the horizontal plane. The aim of this article was to describe and analyze the key parameters that need to be taken into account when transporting bulk materials by belt conveyors fitted with profile conveyor belts.

This paper describes a laboratory device that simulates a section of a conveyor belt between two adjacent transverse protrusions. A strain gauge load sensor PW2G-C3 with a measuring range of 0 ÷ 12 kg is attached to the folding plate of the experimental device, which senses the magnitude of the applied compressive force of the bulk material (river gravel with a grain size of 4 mm ÷ 8 mm) on the transverse protrusion at different inclination angles of the folding plate of the laboratory device.

From the measured pressure force acting on the transverse protrusion, the known value of the inclination angle of the folding plate, and the gravity of the bulk material, the shear friction coefficient in the contact area of the material grains with the surface (Plexi or rubber) of the folding plate is analytically calculated.

If the conveyor belt is equipped with a profile conveyor belt uniformly filled with bulk material in the horizontal section, then when the conveyor belt is guided along the inclined section of the conveyor belt, the material grains do not spill over the transverse protrusions of a lower height than the height of the bulk material layer carried on the working surface of the profile conveyor belt. This fact can be expressed by the fact that the coefficient of the internal friction angle $f_{2}$ [−] (the coefficient of shear friction between individual grains of bulk material on a plane of height $h_{R}$ [m] away from the conveyor belt surface) is higher than the coefficient of shear friction between the material grains $f_{1}$ [−] (the coefficient of the external friction angle) and the conveyor belt working surface.

Overfilling the layer of material grains located above the plane of the height $h_{R}$ [m] of the transverse protrusion occurs when the angle of inclination of the profile belt becomes $\delta_{1k}\geq \mathrm{atan}\left(f_{2}\right)\;[\deg]$.

The protrusions on the working surface of profile conveyor belts are usually realized by manufacturers in the shape of a V, where one arm of the “V” protrusion is at an angle $\alpha_{1}$ [deg] with the longitudinal axis of the conveyor belt. In this paper, the calculation of the angle of inclination of the protrusion arm is performed, at which there is no relative movement of the bulk material in the lateral direction of the conveyor belt (material grains do not spill over the lateral edge of the profile conveyor belt) at the angle of inclination of transport.

## Figures and Tables

**Table 2:** Angle of protrusion inclination $\alpha_{1}$ [deg] and coefficient k [−] at the transport inclination angle $\delta_{1}$ [deg] and shear friction coefficient f_1_ = 0.25.

Shear Friction Coefficient f_1_ = 0.25										
δ_1_ [deg]	18	20	22	24	26	28	30	32	34	36
$\alpha_{1}$ [deg] ^1^	27.68	34.18	39.07	42.96	46.14	48.83	51.12	53.13	54.89	56.46
k [−]	0.769	0.687	0.619	0.562	0.513	0.47	0.433	0.4	0.371	0.344

^1^ see Figure 4.

**Table 3:** Measured values $F_{0M\left(\delta_{1}\right),k}\;[N]$, $F_{FM\left(\delta_{1}\right),k}\;[N]$, and $F_{M\left(\delta_{1}\right),k}\;[N]$ at transport inclination angle $\delta_{1}$ = 18 deg and 23 deg and protrusion inclination angle α = 0 deg.

δ_1_	[deg]	18						23					
k	G	F_0M(18),k_	F_FM(18),k_	F_F(18),k_	F_M(18),k_	F_(18,0),k_	f_1P(18,0),k_	F_0M(23),k_	F_FM(23),k_	F_F(23),k_	F_M(23),k_	F_(23,0),k_	f_1P(23,0),k_
	[N]	[N]	[N]	[N]	[N]	[N]	[−]	[N]	[N]	[N]	[N]	[N]	[−]
1	70	−0.41 ^1^	0.28 ^1^	0.69 ^1^	2.98 ^1^	3.11 ^1^	0.28	−0.38 ^2^	1.27 ^2^	1.65 ^2^	8.14 ^2^	7.25 ^2^	0.31
2	70	−0.28	0.29	0.57	3.17	3.16	0.28	−0.26	1.13	1.39	8.43	7.56	0.31
3	70	−0.36	0.28	0.64	3.04	3.12	0.28	−0.31	1.18	1.49	7.96	7.09	0.31
					f_1P(18,0)AM_ =		0.28				f_1P(23,0)AM_ =		0.31
					κ_(5%,3)18_ =		±0.00				κ_(5%,3)23_ =		±0.00

^1^ see Figure 7a, ^2^ see Figure 7b.

**Table 4:** Measured values $F_{0M\left(\delta_{1}\right),k}\;[N]$, $F_{FM\left(\delta_{1}\right),k}\;[N]$, $F_{M\left(\delta_{1}\right),k}\;[N]$ at transport inclination angle $\delta_{1}$ = 18 deg and 23 deg and protrusion inclination angle $\alpha_{1}=90\;\deg$.

δ_1_	[deg]	28						32					
k	G	F_0M(28),k_	F_FM(28),k_	F_F(28),k_	F_M(28),k_	F_(28,0),k_	f_1P(28,0),k_	F_0M(32),k_	F_FM(32),k_	F_F(32),k_	F_M(32),k_	F_(32,0),k_	f_1P(32,0),k_
	[N]	[N]	[N]	[N]	[N]	[N]	[−]	[N]	[N]	[N]	[N]	[N]	[−]
1	70	−0.41 ^1^	1.37 ^1^	1.78 ^1^	12.69 ^1^	11.73 ^1^	0.34	−0.29 ^2^	1.62 ^2^	1.91 ^2^	19.43 ^2^	18.10 ^2^	0.32
2	70	−0.39	1.61	2.00	13.91	12.69	0.33	−0.40	1.71	2.11	18.81	17.50	0.33
3	70	−0.37	1.28	1.65	13.78	12.87	0.32	−0.32	1.69	2.01	18.76	17.39	0.33
					f_1P(28,0)AM_ =		0.33				f_1P(32,0)AM_ =		0.33
					κ_(5%,3)28_ =		±0.02				κ_(5%,3)32_ =		±0.01

^1^ see Figure 8, ^2^ see Figure 9a.

**Table 5:** Measured values $F_{0M\left(38\right),k}\;[N]$, $F_{FM\left(38\right),k}\;[N]$, $F_{M\left(38\right),k}\;[N]$ at transport inclination angle $\delta_{1}$ = 38 deg and protrusion inclination angle $\alpha_{1}=90\;\deg$.

δ_1_	[deg]	38					
k	G	F_0M(38),k_	F_FM(38),k_	F_F(38),k_	F_M(38),k_	F_(38,0),k_	f_1P(38,0),k_
	[N]	[N]	[N]	[N]	[N]	[N]	[−]
1	70	−0.03 ^1^	1.94 ^1^	1.97 ^1^	26.87 ^1^	24.96 ^1^	0.33
2	70	−0.09	1.98	2.07	26.90	25.01	0.33
3	70	−0.06	1.96	2.02	26.84	24.94	0.33
					f_1P(38,0)AM_ =		0.33
					κ_(5%,3)38_ =		±0.00

^1^ see Figure 9b.

**Table 6:** Measured values $F_{0M\left(\delta_{1}\right),k}\;[N]$, $F_{FM\left(\delta_{1}\right),k}\;[N]$, and $F_{M\left(\delta_{1}\right),k}\;[N]$ at transport inclination angle $\delta_{1}$ = 18 deg and 23 deg and protrusion inclination angle $\alpha_{1}=75\;\deg$.

δ_1_	[deg]	18						23					
k	G	F_0M(18),k_	F_FM(18),k_	F_F(18),k_	F_M(18),k_	F_(18,15),k_	f_1P(18,15),k_	F_0M(23),k_	F_FM(23),k_	F_F(23),k_	F_M(23),k_	F_(23,15),k_	f_1P(23,15),k_
	[N]	[N]	[N]	[N]	[N]	[N]	[−]	[N]	[N]	[N]	[N]	[N]	[−]
1	45	−0.52 ^1^	0.29 ^1^	0.81 ^1^	1.39 ^1^	1.62 ^1^	0.29	−0.31 ^2^	0.48 ^2^	0.79 ^2^	5.57 ^2^	5.40 ^2^	0.29
2	45	−0.52	0.14	0.66	1.66	2.04	0.28	−0.26	0.61	0.87	4.95	4.60	0.31
3	45	−0.50	0.00	0.50	1.38	1.88	0.28	−0.29	0.47	0.76	4.85	4.67	0.31
					f_1P(18,15)AM_ =		0.28				f_1P(23, 15)AM_ =		0.30
					κ_(5%,3)18_ =		±0.01				κ_(5%,3)23_ =		±0.02

^1^ see Figure 10a, ^2^ see Figure 10b.

**Table 7:** Measured values $F_{0M\left(\delta_{1}\right),k}\;[N]$, $F_{FM\left(\delta_{1}\right),k}\;[N]$, and $F_{M\left(\delta_{1}\right),k}\;[N]$ at transport inclination angle $\delta_{1}$ = 28 deg and 32 deg and protrusion inclination angle $\alpha_{1}=75\;\deg$.

δ_1_	[deg]	28						32					
k	G	F_0M(28),k_	F_FM(28),k_	F_F(28),k_	F_M(28),k_	F_(28,15),k_	f_1P(28,15),k_	F_0M(32),k_	F_FM(32),k_	F_F(32),k_	F_M(32),k_	F_(32,15),k_	f_1P(32,15),k_
	[N]	[N]	[N]	[N]	[N]	[N]	[−]	[N]	[N]	[N]	[N]	[N]	[−]
1	45	−0.08 ^1^	1.22 ^1^	1.30 ^1^	9.95 ^1^	8.81 ^1^	0.31	0.08 ^2^	1.41 ^2^	1.33 ^2^	13.43 ^2^	12.02 ^2^	0.31
2	45	−0.08	0.77	0.85	9.49	8.80	0.31	0.11	1.74	1.63	13.41	11.67	0.32
3	45	−0.09	1.29	1.38	9.67	8.47	0.32	0.13	1.61	1.48	13.07	11.46	0.32
					f_1P(28,15)AM_ =		0.31				f_1P(32, 15)AM_ =		0.32
					κ_(5%,3)28_ =		±0.01				κ_(5%,3)32_ =		±0.01

^1^ see Figure 11a, ^2^ see Figure 11b.

**Table 8:** Measured values $F_{0M\left(38\right),k}\;[N]$, $F_{FM\left(38\right),k}\;[N]$, and $F_{M\left(38\right),k}\;[N]$ at transport inclination angle $\delta_{1}$ = 38 deg and protrusion inclination angle $\alpha_{1}=75\;\deg$.

δ_1_	[deg]	38					
k	G	F_0M(38),k_	F_FM(38),k_	F_F(38),k_	F_M(38),k_	F_(38,15),k_	f_1P(38,15),k_
	[N]	[N]	[N]	[N]	[N]	[N]	[−]
1 ^1^	45	−0.23	0.53	0.76	15.83	15.53	0.34
2	45	−0.16	0.69	0.85	15.41	14.88	0.36
3	45	−0.19	0.62	0.81	15.27	14.84	0.36
					f_1P(38,0)AM_ =		0.35
					κ_(5%,3)38_ =		±0.02

^1^ see Figure 12.

**Table 9:** Measured values $F_{0M\left(\delta_{1}\right),k}\;[N]$, $F_{FM\left(\delta_{1}\right),k}\;[N]$, and $F_{M\left(\delta_{1}\right),k}\;[N]$ at transport inclination angle $\delta_{1}$ = 18 deg and 23 deg and protrusion inclination angle $\alpha_{1}=90\;\deg$.

δ_1_	[deg]		18					
k	G		F_0M(18),k_	F_FM(18),k_	F_F(18),k_	F_M(18),k_	F_(18,0),k_	f_1R(18,0),k_
	[N]		[N]	[N]	[N]	[N]	[N]	[−]
1	70		−0.77 ^1^	0.44 ^1^	1.21 ^1^	1.12 ^1^	1.55 ^1^	0.30
2	70		−1.35	0.26	1.61	1.38	2.47	0.29
3	70		−1.61	0.13	1.74	1.32	2.80	0.28
						f_1R(18,0)AM_ =		0.29
						κ_(5%,3)18_ =		±0.02
δ_1_			23					
k		G	F_0M(23),k_	F_FM(23),k_	F_F(23),k_	F_M(23),k_	F_(23,0),k_	f_1R(23,0),k_
		[N]	[N]	[N]	[N]	[N]	[N]	[−]
1		41	−1.31 ^2^	0.02 ^2^	1.33 ^2^	2.85 ^2^	4.14 ^2^	0.31
2		41	−0.76	1.22	1.98	4.03	3.57	0.32
3		41	−0.60	0.48	1.08	2.97	3.09	0.34
						f_1R(23,0)AM_ =		0.32
						κ_(5%,3)23_ =		±0.03

^1^ see Figure 13a, ^2^ see Figure 13b.

**Table 10:** Measured values $F_{0M\left(\delta_{1}\right),k}\;[N]$, $F_{FM\left(\delta_{1}\right),k}\;[N]$, and $F_{M\left(\delta_{1}\right),k}\;[N]$ at transport inclination angle $\delta_{1}$ = 28 deg and 32 deg and protrusion inclination angle $\alpha_{1}=90\;\deg$.

δ_1_	[deg]	28						32					
k	G	F_0M(28),k_	F_FM(28),k_	F_F(28),k_	F_M(28),k_	F_(28,0),k_	f_1R(28,0),k_	F_0M(32),k_	F_FM(32),k_	F_F(32),k_	F_M(32),k_	F_(32,0),k_	f_1R(32,0),k_
	[N]	[N]	[N]	[N]	[N]	[N]	[−]	[N]	[N]	[N]	[N]	[N]	[−]
1	41	−0.85 ^1^	0.22 ^1^	1.07 ^1^	4.02 ^1^	4.65 ^1^	0.40	−0.12 ^2^	0.94 ^2^	1.06 ^2^	5.01 ^2^	4.19 ^2^	0.50
2	41	−1.26	0.04	1.30	4.11	5.33	0.38	−0.43	1.32	1.75	5.75	4.86	0.49
3	41	−0.58	0.76	1.34	4.75	4.57	0.41	−0.15	0.53	0.68	5.96	5.58	0.46
					f_1R(18,0)AM_ =		0.40				f_1R(23,0)AM_ =		0.48
					κ_(5%,3)28_ =		±0.03				κ_(5%,3)32_ =		±0.04

^1^ see Figure 14a, ^2^ see Figure 14b.

**Table 11:** Measured values $F_{0M\left(38\right),k}\;[N]$, $F_{FM\left(38\right),k}\;[N]$, and $F_{M\left(38\right),k}\;[N]$ at transport inclination angle $\delta_{1}$ = 38 deg and protrusion inclination angle $\alpha_{1}=90\;\deg$.

δ_1_	[deg]	38					
k	G	F_0M(38),k_	F_FM(38),k_	F_F(38),k_	F_M(38),k_	F_(38,0),k_	f_1R(38,0),k_
	[N]	[N]	[N]	[N]	[N]	[N]	[−]
1 ^1^	70	−1.27	−0.39	0.88	15.83	17.49	0.46
2	70	−2.83	−0.56	2.27	16.64	20.03	0.47
3	70	−2.32	−0.53	1.79	16.13	18.98	0.48
					f_1R(38,0)AM_ =		0.47
					κ_(5%,3)38_ =		±0.02

^1^ see Figure 15.

**Table 12:** Measured values $F_{0M\left(\delta_{1}\right),k}\;[N]$, $F_{FM\left(\delta_{1}\right),k}\;[N]$, and $F_{M\left(\delta_{1}\right),k}\;[N]$ at transport inclination angle δ_1_ = 23 deg and 28 deg and protrusion inclination angle α_1_ = 75 deg.

δ_1_	[deg]	23						28					
k	G	F_0M(28),k_	F_FM(23),k_	F_F(23),k_	F_M(23),k_	F_(23,15),k_	f_1R(23,15),k_	F_0M(28),k_	F_FM(28),k_	F_F(28),k_	F_M(28),k_	F_(28,15),k_	f_1R(28,15),k_
	[N]	[N]	[N]	[N]	[N]	[N]	[−]	[N]	[N]	[N]	[N]	[N]	[−]
1	41	−1.30 ^1^	−0.54 ^1^	0.76 ^1^	0.45 ^1^	1.75 ^1^	0.38	−1.39 ^2^	0.55 ^2^	1.94 ^2^	4.48 ^2^	5.32 ^2^	0.45
2	41	−0.43	−0.14	0.29	0.77	1.20	0.39	−0.16	0.28	0.44	3.15	3.03	0.45
3	41	−0.96	−0.41	0.55	0.59	1.55	0.38	−0.50	−0.17	0.33	3.61	4.11	0.42
					f_1R(23,15)AM_ =		0.38				f_1R(28,15)AM_ =		0.44
					κ_(5%,3)23_ =		±0.01				κ_(5%,3)28_ =		±0.03

^1^ see Figure 16a, ^2^ see Figure 16b.

**Table 13:** Measured values $F_{0M\left(\delta_{1}\right),k}\;[N]$, $F_{FM\left(\delta_{1}\right),k}\;[N]$, and $F_{M\left(\delta_{1}\right),k}\;[N]$ at transport inclination angle $\delta_{1}$ = 32 deg and 38 deg and protrusion inclination angle $\alpha_{1}=75\;\deg$.

δ_1_	[deg]	32						38					
k	G	F_0M(32),k_	F_FM(32),k_	F_F(32),k_	F_M(32),k_	F_(32,0),k_	f_1R(32,15),k_	F_0M(38),k_	F_FM(38),k_	F_F(38),k_	F_M(38),k_	F_(38,0),k_	f_1R(38,15),k_
	[N]	[N]	[N]	[N]	[N]	[N]	[N]	[N]	[N]	[N]	[N]	[N]	[−]
1	41	−0.15 ^1^	0.45 ^1^	0.60 ^1^	3.79 ^1^	3.49 ^1^	0.52	−1.31	1.04	2.35	8.97	9.24	0.50
2	41	−0.63	1.23	1.86	3.43	2.83	0.54	0.35 ^2^	0.69 ^2^	0.34 ^2^	8.71 ^2^	8.37 ^2^	0.52
3	41	−0.71	1.16	1.87	3.38	2.93	0.54	−0.21	0.13	0.34	9.04	9.12	0.50
					f_1R(32,15)AM_ =		0.53				f_1R(38,15)AM_ =		0.51
					κ_(5%,3)32_ =		±0.02				κ_(5%,3)38_ =		±0.02

^1^ see Figure 17a, ^2^ see Figure 17b.

**Table 14:** Shear friction coefficient $f_{1j\left(\delta_{1},\alpha \right),k}\;\left[-\right]$ of bulk material (river gravel of grain size 4 ÷ 8 mm) on the contact surface of the folding plate inclined at an angle $\delta_{1}$ [deg].

δ_1_ [deg]		18	23	28	32	38
$f_{1P\left(\delta_{1},0\right)\mathrm{AM}}$	[−]	0.28	0.31 ^1^	0.33 ^1^	0.33 ^1^	0.33 ^1^
$f_{1P\left(\delta_{1},15\right)\mathrm{AM}}$		0.28	0.30 ^2^	0.31 ^2^	0.32 ^2^	0.35 ^2^
$f_{1R\left(\delta_{1},0\right)\mathrm{AM}}$		0.29	0.32 ^3^	0.40 ^3^	0.48 ^3^	0.47 ^3^
$f_{1R\left(\delta_{1},15\right)\mathrm{AM}}$		−	0.38 ^4^	0.44 ^4^	0.53 ^4^	0.51 ^4^

see Figure 18 and ^1^ Table 3, Table 4 and Table 5; ^2^ Table 6, Table 7 and Table 8; ^3^ Table 10 and Table 11; ^4^ Table 12 and Table 13.

**Table 15:** Internal friction angle coefficient $f_{2,k}\;\left[-\right]$ (coefficient of shear friction) of bulk material (river gravel of 4 ÷ 8 mm grain size) on the contact surface of the folding plate, inclined at an angle $\delta_{1k}$ [deg].

$\delta_{1k}$	[deg]	29.4 ^1^	27.6	28.1	29.2	27.9		
k		1	2	3	4	5		
$f_{2,k}$	[−]	0.56	0.52	0.53	0.56	0.53	$f_{2,AM}$ = 0.54	κ_(5%,5)_ = 0.03

^1^ see Figure 19b.

## Data Availability

Measured data of effective vibration speed values i_RMS(α,β,m,ne)_ [mm·s^−1^], listed in Table 3, Table 4, Table 5, Table 6, Table 7, Table 8, Table 9, Table 10, Table 11, Table 12 and Table 13 and processed using DEWESoft X software, can be sent in case of interest, by prior written agreement, in *.XLSX (Microsoft Excel) format.
